# Scenario dependence of future changes in climate extremes under 1.5 °C and 2 °C global warming

**DOI:** 10.1038/srep46432

**Published:** 2017-04-20

**Authors:** Zhili Wang, Lei Lin, Xiaoye Zhang, Hua Zhang, Liangke Liu, Yangyang Xu

**Affiliations:** 1 State Key Laboratory of Severe Weather and Key Laboratory of Atmospheric Chemistry of CMA, Chinese Academy of Meteorological Sciences, Beijing, China; 2 School of Atmospheric Sciences and Guangdong Province Key Laboratory for Climate Change and Natural Disaster Studies, Sun Yat-sen University, Guangzhou, China; 3 Chinese Academy of Meteorological Sciences & Center for Excellence in Regional Atmospheric Environment, IUN, CAS, China; 4 Laboratory for Climate Studies, National Climate Center, China Meteorological Administration, Beijing, China; 5 Department of Atmospheric Sciences, Texas A&M University, College Station, Texas, USA; 6 Nanjing University of Information Science and Technology, Nanjing, China

## Abstract

The 2015 Paris Agreement aims to limit global warming below 2 °C and pursue efforts to even limit it to 1.5 °C relative to pre-industrial levels. Decision makers need reliable information on the impacts caused by these warming levels for climate mitigation and adaptation measures. We explore the changes in climate extremes, which are closely tied to economic losses and casualties, under 1.5 °C and 2 °C global warming and their scenario dependence using three sets of ensemble global climate model simulations. A warming of 0.5 °C (from 1.5 °C to 2 °C) leads to significant increases in temperature and precipitation extremes in most regions. However, the projected changes in climate extremes under both warming levels highly depend on the pathways of emissions scenarios, with different greenhouse gas (GHG)/aerosol forcing ratio and GHG levels. Moreover, there are multifold differences in several heavily polluted regions, among the scenarios, in the changes in precipitation extremes due to an additional 0.5 °C warming from 1.5 °C to 2 °C. Our results demonstrate that the chemical compositions of emissions scenarios, not just the total radiative forcing and resultant warming level, must be considered when assessing the impacts of global 1.5/2 °C warming.

Scientists have suggested that substantial changes of the Earth system would occur if the global mean surface temperature exceeds the threshold of 2 °C relative to pre-industrial levels, such as large sea level rise due to a melting of major ice sheets in the Greenland and Antarctic, more frequent occurrence of climate extremes, and massive species extinctions^1^,^2^,^3^.

To limit anthropogenic influences on the Earth’s climate, the parties under the United Nations Framework Convention on Climate Change (UNFCCC) took note of the goal of limiting global warming to less than 2 °C relative to pre-industrial levels in 2009^4^. However, many of the impacts projected for 2 °C warming may exceed the adaptation capacity of the most vulnerable countries, such as small island nations^5^. As such, many countries advocated the aggressive goal of limiting warming to less than 1.5 °C. The Paris Agreement passed at the UNFCCC 21^st^ Conference of the Parties in December 2015. This agreement aims to limit global warming to less than 2 °C and pursue efforts to limit it to 1.5 °C^6^. Great efforts such as deep carbon emission cuts and even carbon capture must be implemented to lower the projected warming by 0.5 °C^7^. We believe that the impacts of such a 0.5 °C warming mitigation at regional scales deserve to be assessed to justify the cost of mitigation.

The public predominantly perceives climate change through the effects of climate extremes, which are closely tied to economic losses and casualties^8^. It would be useful to quantify the benefits of mitigation on reducing climate extremes. Several recent studies assessed various extreme metrics at the end of the 21^st^ century for two Representative Concentration Pathway (RCP) scenarios (RCP8.5 and RCP4.5, effectively 3.5 °C and 2.5 °C)^9^,^10^,^11^,^12^. More recently, Schleussner *et al*.^3^ investigated changes in climate extremes associated with warming levels of 1.5 °C and 2 °C based on the Coupled Model Intercomparison Project Phase 5 (CMIP5) multi-model simulations using a single RCP8.5 scenario. The question addressed in that study was “how much climate extremes can be avoided if we can lower the global warming from 2 °C further down to 1.5 °C”. However, compared to RCP8.5, a lower carbon emission pathway (RCP4.5) could yield a slower warming rate that crosses the 1.5 °C and 2 °C in later decades of the 21^st^ century. Assessment based on RCP4.5 scenario may provide a different answer to the aforementioned question.

Moreover, we recently found that the sensitivity of climate extremes to the global mean warming differed greatly among various forcing agents^13^,^14^, such as greenhouse gases (GHGs) and aerosols. GHGs are the major contributor to global warming, while anthropogenic aerosols have masked some of the warming effects of GHG emissions in the past few decades^15^,^16^. Previous assessment on the target of 1.5 °C or 2 °C often focused on methods of reducing GHG emissions^17^,^18^,^19^,^20^. However, GHGs and aerosols share many common sources, such as emissions from fossil fuel combustions. Therefore, part of future aerosol emissions will inevitably be cut concurrently with GHG emissions. The strengthening of regulations by governments worldwide to improve air quality will also lead to a rapid decline in aerosol emissions^21^,^22^. Aerosol concentrations were projected to largely decrease with their emission reductions, as was shown in RCP scenarios^23^. It was also found that global aerosol emissions compared well to and were at times lower than those in the RCPs over the 21^st^ century, when assuming an extrapolation of current and planned air-pollution legislation without new policies to improve energy access^24^. Stringent pollution controls and clean energy policies can further decrease the global aerosol emissions below the RCP levels^24^. Air-pollutant emissions were also projected to greatly decrease in the Shared Socio-economic Pathways primarily framed within the context of climate change mitigation and adaptation^25^. It is likely that aerosol forcing will exacerbate global warming in the future^13^,^15^,^26^,^27^. Thus, studies on emission scenarios required to limit warming to a certain level must explore various combinations of GHG and aerosol forcing under different measures^18^,^28^, for example, by comparing GHG forcing alone with various proportions of GHG and aerosol forcing.

This study aims to determine the extent to which emissions scenarios, with different GHG levels or forcing compositions, affect the changes in climate extremes between global warming of 1.5 °C and 2 °C. We used three sets of Community Earth System Model, version 1 (CESM1) ensemble simulations. They were the RCP8.5 Large Ensemble (LE) based on the RCP8.5 scenario (a high GHG emission pathway)^29^, the RCP4.5 Medium Ensemble (ME) based on the RCP4.5 scenario (a medium-low GHG emission pathway)^30^, and the RCP8.5 with fixed emissions of aerosols and atmospheric oxidants at the year 2005 levels (RCP8.5_FixA)^31^. However, it should be clarified that these are not scenarios that keep global warming to near 1.5 °C or 2 °C by 2100. If one uses such aggressive mitigation scenarios to analyze the end-of-21^st^-century response, the effect of aerosols might be much smaller according to RCP-like aerosol trajectories. Our results show that changes in climate extremes under both warming levels highly depend on the pathways of emissions scenarios. We demonstrate that the chemical compositions of emissions scenarios, not merely the resultant warming level, must be considered when assessing the impacts of 1.5/2 °C global warming.

## Results

### Time series of surface temperature and climate extremes

**Figure 1:**
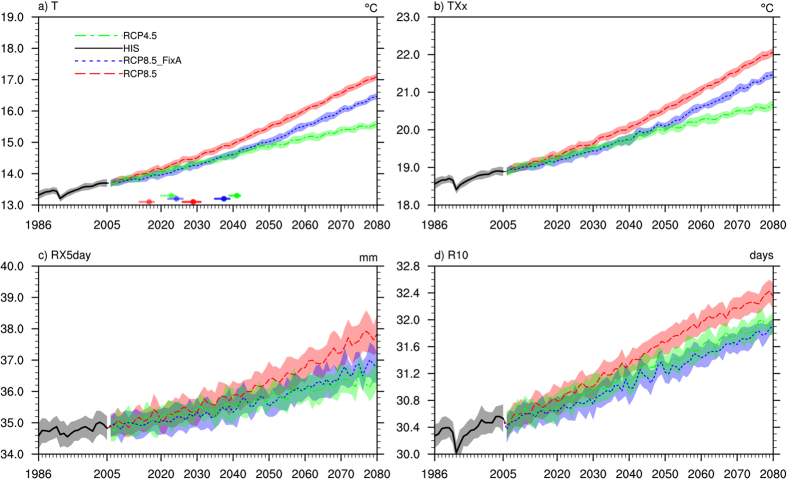
Time series of global annual mean (**a**) surface air temperature, (**b**) TXx, (**c**) RX5 day, and (**d**) R10 under the RCP8.5 (red), RCP4.5 (green), and RCP8.5_FixA (blue) scenarios. The shading represents two standard deviations from three sets of ensemble simulations, respectively. The dots with horizontal lines in (**a**) represent the years in which the global mean surface air temperatures increase by 1.5 °C and 2 °C with a 5% to 95% uncertainty range of the three scenarios.

A large body of evidence suggests that the frequency and intensity of various climate extremes have increased substantially in recent decades due to global warming^32^,^33^. These increases are expected to continue with future warming^34^. Our results also indicate that the global annual averaged surface air temperature and climate extremes, including the monthly maximum of daily maximum temperature (TXx), monthly maximum consecutive 5-day precipitation (RX5 day), and annual number of days with daily precipitation more than 10 mm (R10), in the RCP8.5, RCP4.5 and RCP8.5_FixA scenarios all increase with time (Fig. 1). The increases in these quantities in RCP8.5 are larger than those in RCP8.5_FixA, because the aerosol cooling masks more GHG warming in RCP8.5_FixA. The average time intervals between 1.5 °C and 2 °C global warming are 12, 13, and 18 years in RCP8.5, RCP8.5_FixA, and RCP4.5, respectively (Fig. 1a). The faster warming rate in RCP8.5 leads to an earlier crossing of the 1.5/2 °C threshold.

### Changes in temperature extremes under 1.5/2 °C warming

The extreme temperature index TXx captures the monthly hottest day. Our results show that TXx significantly increases worldwide under a 2 °C warming in all three examined scenarios but exhibits inhomogeneous spatial patterns (Supplementary Fig. S1). TXx increases by >2 °C in West Europe and eastern North America. Increasing warming by 0.5 °C (from 1.5 °C to 2 °C) leads to an increase in TXx of 0.6–1 °C in most of Europe and North America (Supplementary Fig. S1). The spatial pattern of TXx change is largely consistent with several previous results^3^,^35^.

**Figure 2:**
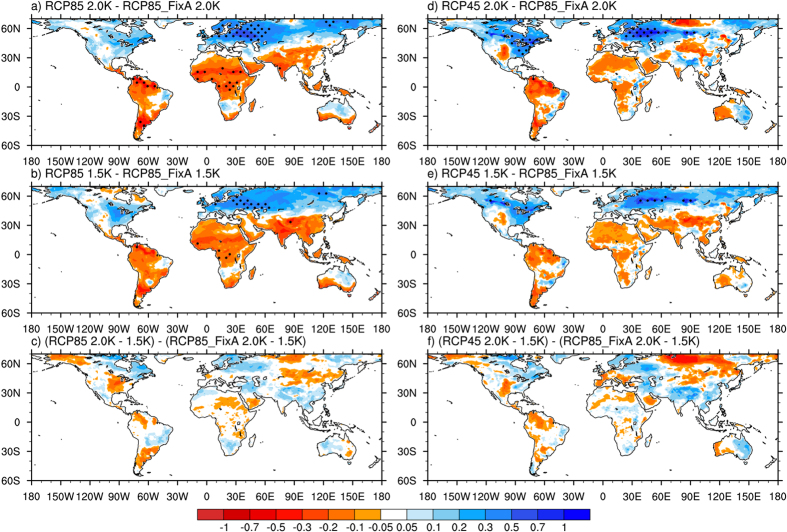
Spatial distributions of differences of changes in TXx under 1.5 °C and 2 °C warming and an additional 0.5 °C warming from 1.5 °C to 2 °C among the RCP8.5, RCP4.5, and RCP8.5_FixA scenarios (unit: °C). The dots represent significance at ≥95% confidence level from a two-sided t-test. Maps were generated using NCAR Command Language (The NCAR Command Language (Version 6.3.0) [Software]. (2013). Boulder, Colorado: UCAR/NCAR/CISL/TDD. http://www.ncl.ucar.edu/).

There are significant differences in the changes in TXx among the three scenarios under 1.5 °C and 2 °C warming (Fig. 2a,b,d,e). The increases in TXx under RCP8.5 and RCP4.5 (i.e., combined impact of GHG and aerosol forcings) are larger than those under RCP8.5_FixA (i.e., the impact of GHG forcing alone) in most mid-latitudes of the Northern Hemisphere (NH). In particular, the maximum difference exceeds 1 °C in western Russia and eastern USA between the RCP4.5 and RCP8.5_FixA simulations. This agrees with our earlier results^13^, which suggested that aerosol forcing had a slightly larger effect on temperature extremes over East Asia than did GHG forcing when normalized by the global mean surface temperature changes. The larger sensitivity may be because aerosols can affect the temperature by directly altering both solar radiation and cloud properties^15^. However, the increases in TXx under RCP8.5 and RCP4.5 are less than those under RCP8.5_FixA in South America, Africa, and South Asia.

### Changes in precipitation extremes under 1.5/2 °C warming

Our previous analysis^14^ has suggested that aerosol forcing produces a larger effect on future precipitation extremes than does GHG forcing when normalized by global mean surface temperature changes. This can be attributed to several mechanisms. First, changes in precipitation are constrained by atmospheric radiative cooling. Atmospheric heating caused by GHGs suppresses the response of precipitation to warming relative to scattering aerosols. Second, the vertical structure of aerosol radiative forcing (i.e., positive forcing at the top of the atmosphere and negative forcing in the atmosphere) results in a larger hydrologic cycle response compared to GHG forcing. Third, the non-uniform distribution of aerosol forcing can alter the ocean/land partitioning of precipitation via dynamic responses to forcing. Finally, aerosols can affect rainfall by directly changing cloud microphysics.

**Figure 3:**
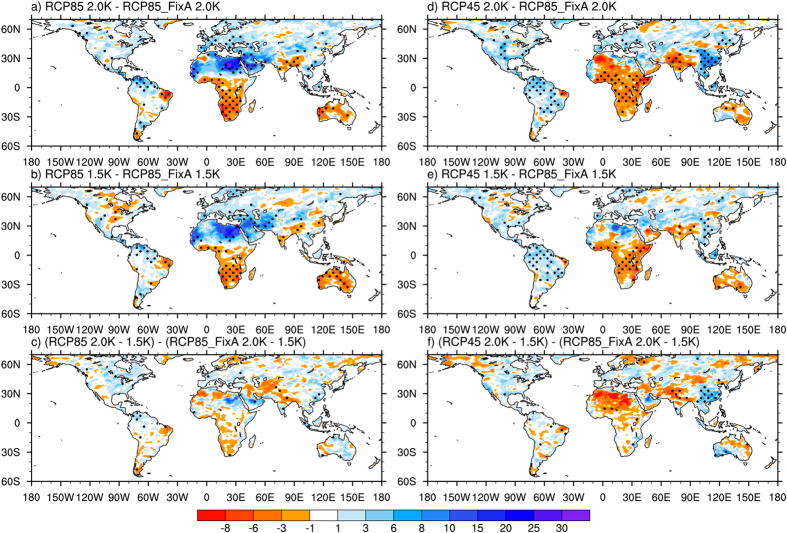
Spatial distributions of differences of changes in RX5 day under 1.5 °C and 2 °C warming and an additional 0.5 °C warming from 1.5 °C to 2 °C among the RCP8.5, RCP4.5, and RCP8.5_FixA scenarios (unit: %). The dots represent significance at ≥95% confidence level from a two-sided t-test. Maps were generated using NCAR Command Language (The NCAR Command Language (Version 6.3.0) [Software]. (2013). Boulder, Colorado: UCAR/NCAR/CISL/TDD. http://www.ncl.ucar.edu/).

The index RX5 day can be used as an indicator of flooding. Similar to the changes in TXx, RX5 day increases in most land areas under 2 °C warming. Significant increases occur in mid-latitudes of the NH, eastern China, Africa, and central South America, with the maximum exceeding 20% (Supplementary Fig. S2). An additional warming by 0.5 °C results in varying degrees of increase in RX5 day depending on the region. The increases are prominent in eastern China and Russia, India, and central Africa, with the maximum being >10% (Supplementary Fig. S2). This is consistent with the previous result based on the CMIP5 multimodel simulations^3^. The changes in RX5 day exhibit strong scenario dependence in most regions under both warming levels (Fig. 3a,b,d,e). For example, differences in the changes in RX5 day between the RCP8.5 and RCP8.5_FixA simulations range from 8% to 30% in northern Africa, the Mediterranean, West Asia, and northern South America. The corresponding differences between the RCP4.5 and RCP8.5_FixA simulations exceed 8% over most of eastern China, Southeast Asia, and Central America.

**Figure 4:**
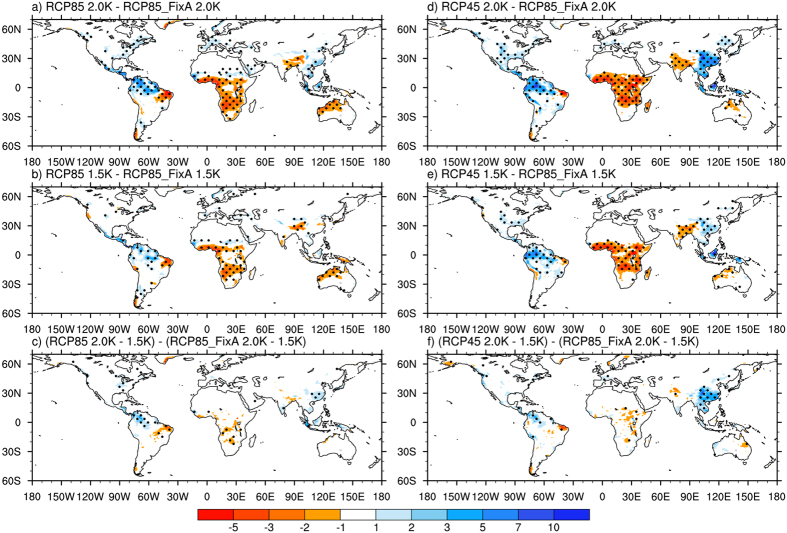
Spatial distributions of differences of changes in R10 under 1.5 °C and 2 °C warming and an additional 0.5 °C warming from 1.5 °C to 2 °C among the RCP8.5, RCP4.5, and RCP8.5_FixA scenarios (unit: days). The dots represent significance at ≥95% confidence level from a two-sided t-test. Maps were generated using NCAR Command Language (The NCAR Command Language (Version 6.3.0) [Software]. (2013). Boulder, Colorado: UCAR/NCAR/CISL/TDD. http://www.ncl.ucar.edu/).

R10 is a more loosely defined extreme precipitation index than is RX5 day. It may be more associated with flooding and other hazards^36^. The sensitivity of precipitation extremes indices in response to different forcings has a larger difference if the indices were loosely defined (i.e., “less extreme”)^14^. R10 increases by 3 to 10 days in East Asia, Southeast Asia, eastern North America, and central South America and Africa under 2 °C warming. However, it decreases in northern South America, Latin America, India, and some coastal regions of central Africa (Supplementary Fig. S3). An additional 0.5 °C warming leads to increases in R10 in eastern China, Southeast Asia, and central Africa, but reductions in northern South America and Latin America (Supplementary Fig. S3). The changes in R10 under both warming levels vary notably in many of the tropical lands among the three scenarios (Fig. 4a,b,d,e). The differences in the projected R10 among the scenarios are comparable to or larger than the changes in R10 in specific scenarios under a given warming level. Compared to RCP8.5_FixA, the changes in R10 are greater in northern South America, Latin America, eastern China, and Southeast Asia under RCP8.5 and RCP4.5. However, the corresponding values are lower in central and southern Africa, South Asia, eastern Brazil, and western Australia under both scenarios.

### Changes in climate extremes due to an additional 0.5 °C warming

**Figure 5:**
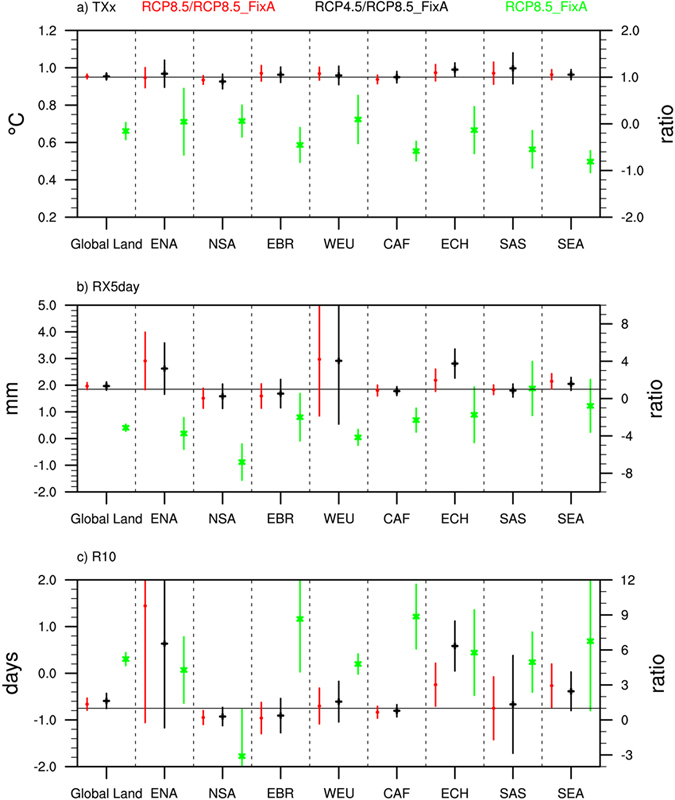
Global- and regional-land average changes of (**a**) TXx (unit: °C), (**b**) RX5 day (unit: mm), and (**c**) R10 (unit: days) caused by an additional 0.5 °C warming from 1.5 °C to 2 °C in the RCP8.5_FixA scenario (Green) (left y-axis) and ratios of their changes in the RCP8.5 (Red) and RCP4.5 (Black) to RCP8.5_FixA scenarios (right y-axis). The error bar denotes two standard deviation, respectively. ENA (East North America, 30 °N–55 °N, 45 °W–100 °W), NSA (Northwest South America, 0–12 °N, 47 °W–80 °W), EBR (East Brazil, 30 °N–55 °N, 35 °W–50 °W), WEU (West Europe, 37 °N–69 °N, 10 °W–43 °E), CAF (Central Africa, 18 °S–12 °N, 15 °W–50 °E), ECH (East China, 20 °N–40 °N, 105 °E–125 °E), SAS (South Asia, 8 °N–29 °N, 70 °E–93 °E), and SEA (Southeast Asia, 10 °S–20 °N, 95 °E–153 °E).

The projected changes in climate extremes caused by an additional 0.5 °C warming, i.e., from 1.5 °C to 2 °C, vary among the three scenarios (Figs 2, 3, 4 and 5). The differences in the changes in TXx due to an additional 0.5 °C increase among the scenarios mainly appear in regions with high levels of aerosol emissions, including China and South Asia (Fig. 5a). The increases in TXx in those areas under RCP8.5 and RCP4.5 are about 20% larger than that under RCP8.5_FixA. The changes in precipitation extremes show more substantial differences among the three scenarios than temperature extreme (Fig. 5b,c). The increases in global land average RX5 day due to 0.5 °C additional warming under RCP8.5 and RCP4.5 are both 30% larger than those under RCP8.5_FixA. The corresponding values are 37% and 67% for R10. The increases in these precipitation extremes averaged over several heavily polluted regions, such as East North America, East China, West Europe, and Southeast Asia, under RCP8.5 and RCP4.5 are several times greater than those under RCP8.5_FixA. However, there are great uncertainties for the changes in these daily-based extreme indices. Also, the differences in emissions scenarios are more likely to affect the magnitudes of changes in climate extremes under a given warming level rather than the distributions of them (Table 1).

## Discussion

Similar to the previous study, we find that increasing warming by 0.5 °C (from 1.5 °C to 2 °C) leads to significant increases in temperature and precipitation extremes in most regions. The increase of climate extremes occurrence helps to justify the cost of climate mitigation. However, a systematic quantitative assessment of the climate benefits of limiting the warming from 2 °C to 1.5 °C would be needed. Currently available assessments are limited in that they often considered the climate model simulations under a single scenario pathway.

Our results show for the first time that the differences in emissions scenarios used by climate models can significantly affect the changes in climate extremes under 1.5 °C and 2 °C warming. The scenario dependence of the changes in precipitation extremes is larger than that in temperature extremes. Note that this study is based on a single climate model, although large ensemble simulations are used. We must acknowledge that the sensitivity of climate extremes to GHG or aerosol forcing can be different among different models.

**Figure 6:**
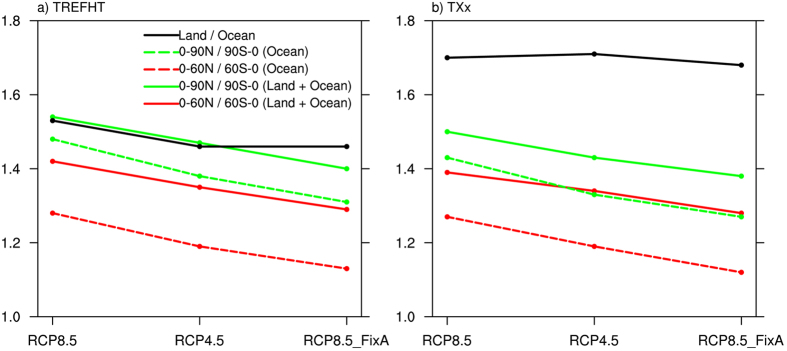
The land-ocean and north-south contrasts of changes in (**a**) 2-m air temperature and (**b**) TXx caused by an additional 0.5 °C warming from 1.5 °C to 2 °C in the RCP8.5, RCP4.5, and RCP8.5_FixA simulations.

The fact that changes in temperature and precipitation extremes have different regional patterns in the three scenarios is directly related to the distinct spatial pattern of surface warming (despite having the same global average of 0.5 °C) (Fig. 6). Here we only show the change in TXx due to a similar situation between temperature and precipitation extremes. A robust feature of the projected surface warming pattern in all cases considered here is the north-south asymmetry, with a faster warming in the NH than the Southern Hemisphere (Fig. 6a). Such a latitudinal asymmetry is due to two possible reasons. (1) A larger land fraction in the NH has a smaller heat capacity^37^. This is supported by the fact that the asymmetry is larger when both land and ocean temperatures are considered rather than ocean temperature alone. (2) The upwelling water in the Southern Ocean around the Antarctic increases the heat mixing into the deep ocean^38^. This is also supported by our results that the asymmetry is larger when the 90 °S–90 °N temperature is considered rather than 60 °S–60 °N alone. Such a north-south asymmetry of warming is expected to weaken when given more time for the climate system, particularly the deep ocean, to adjust. Consistently, we find that the north-south asymmetry is largest in the fastest warming RCP8.5 case with the shortest time interval between 1.5 °C and 2 °C warming. The asymmetry is the lowest for RCP8.5_FixA. Not surprisingly, this is because the lack of aerosol forcing in the NH should have enhanced the NH warming on top of the inertia mechanism discussed above. It’s very interesting that the land-ocean contrast is even larger in TXx than mean temperature (Fig. 6b). This points to the importance of considering this fundamental asymmetry and its regional implication of extreme projections.

Our results demonstrate that the emissions scenario pathway, both regarding GHG/aerosol ratio and GHG levels, must be considered when assessing the impacts of global warming mitigation. Such a non-linearity of regional climate response to the same global warming interval has recently been noted^39^. This study motivates other impact assessment studies to be more cautious in interpreting the 1.5/2 °C warming simulations under a single scenario, such as RCP8.5. Ideally, to evaluate the climate impact of stabilizing the warming at 1.5/2 °C, 21^st^-century model simulations based on the “real” carbon-neutrality scenarios, such as those proposed in Sanderson *et al*.^7^ would be needed.

## Methods

### Climate model

We used the CESM1, a fully-coupled Earth system model, with a 0.9° (latitude) ×1.25° (longitude) spatial resolution for the atmosphere^40^. CESM1 includes the major anthropogenic forcing agents, like GHGs, stratospheric and tropospheric ozone, and aerosols. The three-mode modal aerosol model that can predict the number and mass concentrations of internally mixed aerosols has been implemented in the model^41^. The model includes the physical processes of aerosol-radiation and aerosol-stratiform cloud interactions^42^,^43^,^44^.

### Simulations

We used three sets of CESM1 ensemble simulations:

(1) RCP8.5 LE^29^. The RCP8.5 LE consists of 30-member ensembles integrated from 1920 to 2100 in term of the RCP8.5 scenario (a high GHG emission pathway)^45^. Each member uses the same evolutions of GHG and aerosol forcings but starts from slightly different atmospheric initial conditions. The emissions of various aerosol species from 2000 to 2100 in the RCP8.5 scenario are shown in Supplementary Fig. S4.

(2) RCP4.5 ME^30^. The RCP4.5 ME consists of 15-member ensembles from 2005 to 2080. It is similar to RCP8.5 LE but follows the RCP4.5 scenario (a medium-low GHG emission pathway)^46^.

(3) RCP8.5_FixA^31^. These 15-member ensemble simulations (2005–2100) also use the forcing from the RCP8.5, but all emissions of aerosols and atmospheric oxidants are fixed at the year 2005 levels.

All simulation data used here are available at https://www.earthsystemgrid.org/dataset/ucar.cgd.ccsm4.output.html. Following the method in Schleussner *et al*.^3^, we selected a reference period of 1986–2005, when the mean temperature was 0.6 °C warmer than pre-industrial levels. Next, for each simulation, we extracted data for periods during which the consecutive 20-year average global mean surface temperature increased by 0.9 °C and 1.4 °C relative to the reference period to represent increases of 1.5 °C and 2 °C above pre-industrial levels, respectively. Then, we assessed the differences between the extracted 20-year data and reference data to determine the changes in climate extremes. The warming of 1.5 °C and 2 °C in RCP8.5 and RCP4.5 was due to the combined impact of GHG and aerosol forcings, but the GHG forcing contributed a larger fraction in RCP8.5 (i.e., a smaller fractional contribution of aerosol forcing) because of the similar aerosol forcing pathway under both scenarios^47^. However, the warming in RCP8.5_FixA was only caused by the GHG forcing.

### Climate extremes indices

We examined three extreme indices based on daily temperature and precipitation data, as described by the Expert Team for Climate Change Detection and Indices^32^, that included the TXx, RX5 day, R10.

## Additional Information

**How to cite this article:** Wang, Z. *et al*. Scenario dependence of future changes in climate extremes under 1.5 °C and 2 °C global warming. *Sci. Rep.* **7**, 46432; doi: 10.1038/srep46432 (2017).

**Publisher's note:** Springer Nature remains neutral with regard to jurisdictional claims in published maps and institutional affiliations.

## Supplementary Material

Supplementary Information

## Figures and Tables

**Table 1:** The pattern correlation coefficients of changes in climate extremes caused by an additional 0.5 °C warming from 1.5 °C to 2 °C among the scenarios.

	TXx		RX5 day		R10	
	RCP8.5	RCP4.5	RCP8.5	RCP4.5	RCP8.5	RCP4.5
RCP8.5_FixA	0.84	0.7	0.65	0.48	0.74	0.65
RCP8.5	—	0.8	—	0.51	—	0.78

All results are significant at the 99% confidence level.
