# Supplementary material for: Scenario dependence of future changes in climate extremes under 1.5 °C and 2 °C global warming
Source: Sci Rep. 2017 Apr 20;7:46432. doi: 10.1038/srep46432 (PMC5397837; doi:10.1038/srep46432)
Supplement: Supplementary Information [file srep46432-s1.pdf]

Scenario dependence of future changes in climate extremes under 1.5 ℃ and 2 ℃ global warming

Zhili Wang[1*], Lei Lin[2*], Xiaoye Zhang[3], Hua Zhang[4], Yangyang Xu[5] & Liangke Liu[6]

[1]State Key Laboratory of Severe Weather and Key Laboratory of Atmospheric Chemistry of CMA, Chinese Academy of Meteorological Sciences, Beijing, China.

[2]Department of Atmospheric Sciences, Sun Yat-sen University, Guangzhou, China.

[3]Chinese Academy of Meteorological Sciences & Center for Excellence in Regional Atmospheric Environment, IUN, CAS, China.

[4]Laboratory for Climate Studies, National Climate Center, China Meteorological Administration, Beijing, China.

[5]Department of Atmospheric Sciences, Texas A&M University, College Station, Texas, USA.

[6]Nanjing University of Information Science and Technology, Nanjing, China.

[*]Corresponding author: L.L. (email: linlei3@mail.sysu.edu.cn) and Z.W. (email: wangzl@camscma.cn).

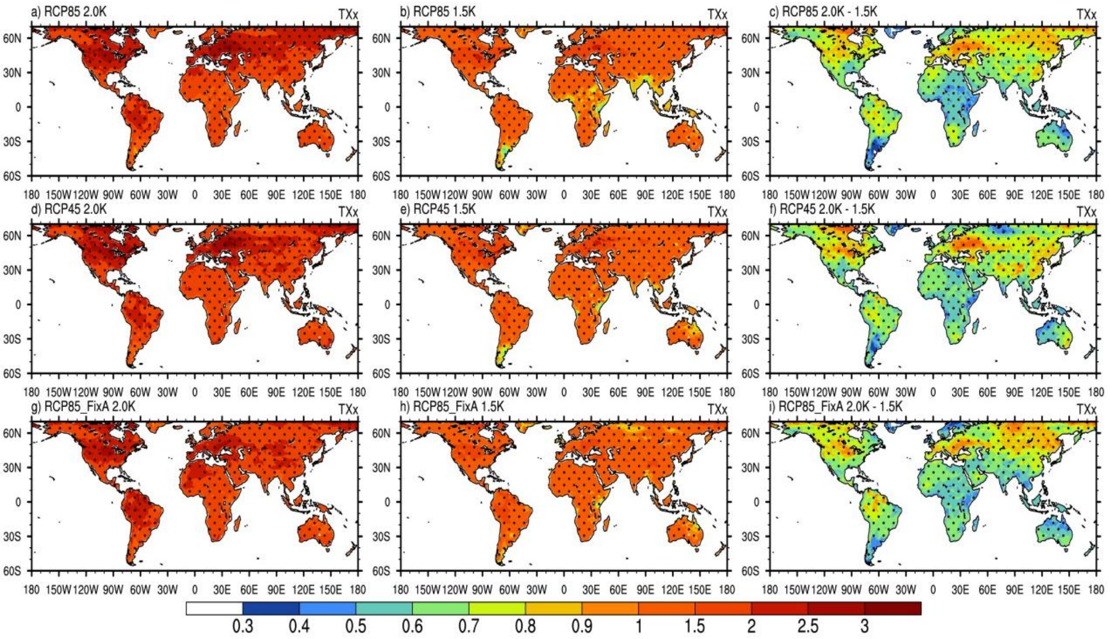

Supplementary Figure S1. Spatial distributions of changes in TXx under 1.5 ℃ and 2 ℃ warming and the difference between the two warming levels in the RCP8.5, RCP4.5, and RCP8.5_FixA scenarios (unit: ℃). The dots represent significance at ≥ 95% confidence level from a two-sided t-test. Maps were generated using NCAR Command Language (The NCAR Command Language (Version 6.3.0) [Software]. (2013). Boulder, Colorado: UCAR/NCAR/CISL/TDD. http://www.ncl.ucar.edu/).

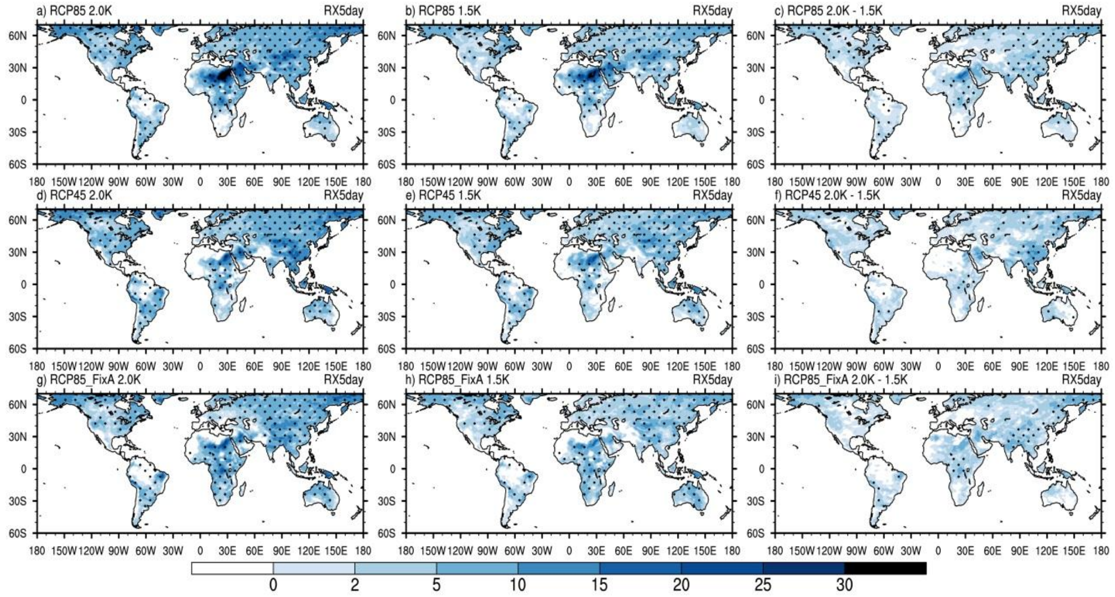

Supplementary Figure S2. Spatial distributions of changes in RX5day under 1.5 ℃ and 2 ℃ warming and the difference between the two warming levels in the RCP8.5, RCP4.5, and RCP8.5_FixA scenarios (unit: %). The dots represent significance at ≥ 95% confidence level from a two-sided t-test. Maps were generated using NCAR Command Language (The NCAR Command Language (Version 6.3.0) [Software]. (2013). Boulder, Colorado: UCAR/NCAR/CISL/TDD. http://www.ncl.ucar.edu/).

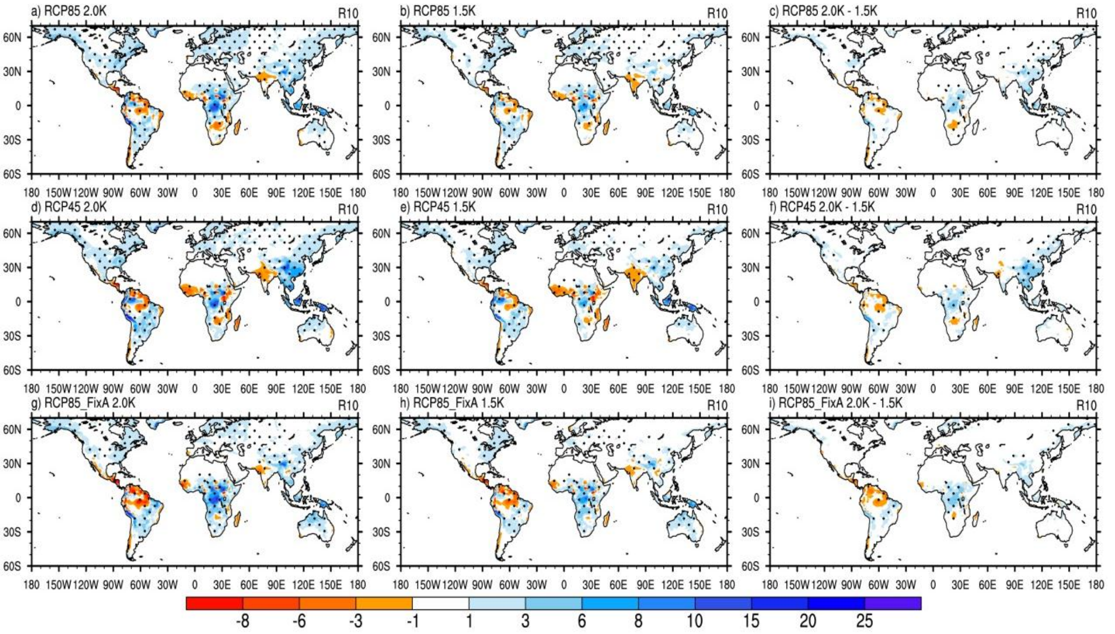

Supplementary Figure S3. Spatial distributions of changes in R10 under 1.5 ℃ and 2 ℃ warming and the difference between the two warming levels in the RCP8.5, RCP4.5, and RCP8.5_FixA scenarios (unit: days). The dots represent significance at ≥ 95% confidence level from a two-sided t-test. Maps were generated using NCAR Command Language (The NCAR Command Language (Version 6.3.0) [Software]. (2013). Boulder, Colorado: UCAR/NCAR/CISL/TDD. http://www.ncl.ucar.edu/).

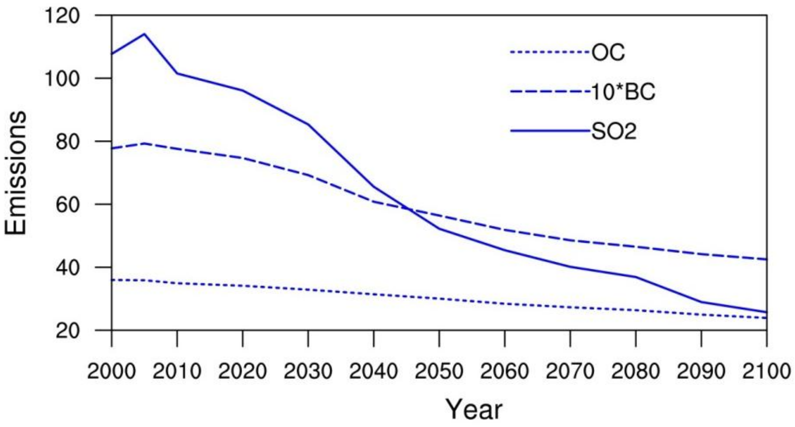

Supplementary Figure S4. SO_2 (unit: Tg yr^{-1}), BC and OC (units: TgC yr^{-1}) emissions from 2000 to 2100 in the RCP8.5 scenario.
